# A psychometric appraisal of the DREEM

**DOI:** 10.1186/1472-6920-12-2

**Published:** 2012-01-12

**Authors:** Sean M Hammond, Margaret O'Rourke, Martina Kelly, Deirdre Bennett, Siun O'Flynn

**Affiliations:** 1 School of Applied Psychology, University College Cork, College road, Cork, Ireland; 2 School of Medicine, University College Cork, College road, Cork, Ireland

## Abstract

**Background:**

The quality of the Educational environment is a key determinant of a student centred curriculum. Evaluation of the educational environment is an important component of programme appraisal. In order to conduct such evaluation use of a comprehensive, valid and reliable instrument is essential. One of most widely used contemporary tools for evaluation of the learning environment is the Dundee Ready Education Environment Measure (DREEM). Apart from the initial psychometric evaluation of the DREEM, few published studies report its psychometric properties in detail. The aim of this study was to examine the psychometric quality of the DREEM measure in the context of medical education in Ireland and to explore the construct validity of the device.

**Methods:**

239 final year medical students were asked to complete the DREEM inventory. Anonymised responses were entered into a database. Data analysis was performed using PASW 18 and confirmatory factor analysis performed.

**Results:**

Whilst the total DREEM score had an acceptable level of internal consistency (alpha 0.89), subscale analysis shows that two subscales had sub-optimal internal consistency. Multiple group confirmatory factor analysis (using Fleming's indices) shows an overall fit of 0.76, representing a weak but acceptable level of fit. 17 of the 50 items manifest fit indices less than 0.70. We sought the best fitting oblique solution to the 5-subscale structure, which showed large correlations, suggesting that the independence of the separate scales is open to question.

**Conclusions:**

There has perhaps been an inadequate focus on establishing and maintaining the psychometric credentials of the DREEM. The present study highlights two concerns. Firstly, the internal consistency of the 5 scales is quite variable and, in our sample, appears rather low. Secondly, the construct validity is not well supported. We suggest that users of the DREEM will provide basic psychometric appraisal of the device in future published reports.

## Background

In 1998, the World Federation for Medical Education highlighted the learning environment as one of the targets for the evaluation of medical education programmes [1]. It is widely agreed among medical educators that the effects of the educational environment, both academic and clinical, are important determinants of medical students attitudes, knowledge, skills, progression and behaviours [2,3]. Evaluation of the educational environment at both academic and clinical sites is key to the delivery of a high quality, student centred curriculum [4]. In order to conduct such evaluation across many sites, specialties and student groups use of a comprehensive, valid and reliable instrument is essential.

Over the last 4 decades educators and researchers have attempted to define and measure the medical education environment [5-11] and the most widely used contemporary development is almost certainly the Dundee Ready Education Environment Measure (DREEM) [12]. The DREEM is a 50-item measure of students' perceptions of their learning environment resulting in scores on five scales. These are labeled, perception of learning, perception of course organizers, academic self perception, perception of atmosphere and social self perception.

The DREEM has proved itself internationally useful in a variety of healthcare settings [13], such as medical, dental, nursing and chiropractic learning environments [14-17]. It has been used to identify weaknesses in curricula with a view to introducing change [18-21], and has been applied to assess the impact of new curricular interventions [22,23,16]. Its focus on student experience has led to its use in identifying the gap between student expectations and experience [24] and student actual and idealised experience [20]. Furthermore, differences between student experience at different sites within medical schools [25,26] and between students perceptions at different stages of their medical education [22], have also been examined using the DREEM. One important use of the DREEM has been as a utility for international comparisons between medical schools [27,28]. This has allowed medical schools to benchmark the educational environment they are providing [29,30].

Its developers have provided a simple guide to interpreting the scores derived from the DREEM making it an accessible and easy-to-use device for evaluating the learning environment in medical education settings [31]. Nevertheless, the fact that it is very commonly used for cross-national comparisons makes it particularly important that it is subject to close ongoing psychometric scrutiny to protect against cultural bias. If the psychometric properties of a device fluctuate across countries, conclusions based on the scale may actually reflect artifacts due to unreliability and lack of validity. There have been repeated calls for rigorous evaluation of the psychometric properties of measures used cross-nationally [32-34], but they are not commonly applied in educational climate studies.

Apart from the initial psychometric evaluation of the DREEM carried out by its originators [12], few of the published studies report it's psychometric properties in any detail. Two exceptions include studies in Portugal, Greece and Sweden [28,29,35]. Results were mixed showing variable levels of internal consistency for the 5 subscales. In addition, factor analyses did not support the 5-factor structure claimed by the DREEM developers. Both studies concluded that the DREEM had clear value and generalized well across different programs but the psychometric shortcomings exposed by their study do invite further scrutiny.

The present study had the following objectives:

1. To examine the psychometric quality of the DREEM measure in the context of medical education in Ireland.

2. To explore the construct validity of the device.

## Method

### Participants

Two cohorts of medical students in their final year of study at University College Cork were sampled. Cohort 1 comprised those entering their final year in 2007 and cohort 2 comprised those entering in 2008. In addition, a third cohort of graduate entry students was sampled for comparison. The complete sample consisted of 239 students.

#### Materials/Instruments Used

Each participant completed the DREEM along with other self-report measures which were part of a larger stress audit study [36]. The focus of this paper is on the DREEM and it was noted that participants took between five and seven minutes to complete this measure.

#### Procedure

Before proceeding, ethical approval was granted by the Clinical Research Ethics Committee of the Cork Teaching Hospitals.

Each participant in the present study was asked to complete the DREEM which was presented as part of a test battery. The task of completing the DREEM was presented during a timetabled lecture slot where the determinants of self directed learning were explored and consequently this material was relevant and although participants were free to withdraw there was 100% adherence.

### Data Management/Analyses

The completed questionnaire responses were entered into an anonymised database for subsequent analysis. Data analysis was performed using PASW 18 and the confirmatory factor analysis was performed using a customised programme written for a windows platform, by the first author.

## Results

Of the 239 medical students participating in this study, 151 were female and 87 were male (one person did not register their gender). Ages ranged between 17 and 39 with a median of 22. The first cohort comprised final year medical students collected in 2007 (N = 102) the second comprised final year medical students collected in 2008 (N = 99) and the third comprised 1^st ^year Graduate entry medical students collected in 2008 (n = 37).

The basic psychometric properties of the DREEM in our sample are reported in table 1. The means and standard deviations appear well within the expected range of scores. Using the guide to interpreting subscale scores [31], it may be concluded that Cork medical students are relatively positive about all aspects of the course. Only two of the scales (Perception of Learning and Academic Self-perception) manifest an alpha exceeding the widely adopted rule of thumb guide of 0.70 [37]. The total DREEM score, in contrast, appears to manifest an acceptable level of internal consistency.

**Table 1:** Classical Psychometric Properties

Subscale	Mean	**S.D**.	n	α
Learning	30.59	6.38	12	0.78
Course Organisers	29.28	5.34	11	0.69
Academic Self Perception	18.57	4.98	8	0.74
Atmosphere	31.33	6.02	12	0.56
Social Self-Perception	17.55	3.95	7	0.55

Total Score	127.08	21.02	50	0.89

The fact that the subscales lack convincing internal consistency raises concerns about the construct validity of the DREEM. In order to evaluate the putative subscale structure of the questionnaire a multiple group confirmatory factor analysis [38,39], was carried out using the DREEM scoring key as an hypothesis matrix. The results are presented in table 2. In a perfect fit to the proposed 5-factor model each DREEM item will load on only one factor producing a value of unity with loadings on all other factors manifesting as zero. In reality such perfect solutions never arise so, in order to evaluate the degree of fit to the model, indices of fit are used. In this study, Fleming's index is used [40]. This shows the fit for each item (seen in the last column of table 2) and also the degree to which each factor fits the model (seen in the last column). The overall fit is estimated as 0.76. As a signal to noise ratio, Fleming's indices share the logic of Cronbach's alpha and may be interpreted similarly. Therefore, an index of 0.76 represents a weak but acceptable level of fit. Nevertheless, bearing in mind the procrustean basis of the multiple group factor analysis procedure, a higher degree of fit would be expected if the model in question was robust and reliable.

**Table 2:** Factor Pattern following a Confirmatory Multiple Group Factor Analysis

**ITEM**	**FACTORS**					
	**I**	**II**	**III**	**IV**	**V**	**Item** **Fit**
Q1	**0.280**	-0.002	-0.034	0.112	-0.091	0.780
Q7	**0.328**	0.022	0.005	-0.008	0.021	0.991
Q13	**0.343**	0.043	-0.092	-0.076	0.057	0.860
Q16	**0.398**	-0.234	0.006	-0.021	0.099	0.708
Q20	**0.386**	-0.032	0.024	-0.160	0.123	0.778
Q21	**0.369**	-0.128	0.010	0.092	-0.090	0.804
Q24	**0.337**	-0.044	-0.031	0.075	-0.015	0.928
Q25	**0.140**	0.179	-0.056	-0.043	0.015	0.347
Q38	**0.253**	0.012	0.092	-0.018	-0.083	0.803
Q44	**0.267**	-0.083	0.076	0.079	-0.013	0.790
Q47	**0.087**	0.148	0.090	0.014	0.064	0.180
Q48	**0.207**	0.119	-0.089	-0.047	-0.087	0.574
Q2	0.119	**0.268**	-0.063	0.051	0.013	0.774
Q6	0.077	**0.238**	0.046	0.051	-0.078	0.772
Q8	-0.115	**0.450**	-0.103	-0.039	-0.130	0.828
Q9	-0.202	**0.516**	0.016	-0.072	-0.057	0.843
Q18	0.093	**0.302**	-0.013	-0.075	0.019	0.860
Q29	-0.057	**0.326**	0.062	-0.011	0.046	0.920
Q32	0.121	**0.186**	-0.019	0.037	-0.020	0.672
Q37	0.055	**0.229**	0.106	-0.034	0.094	0.684
Q39	-0.122	**0.407**	0.021	-0.084	-0.053	0.868
Q40	0.166	**0.292**	-0.132	-0.020	0.062	0.635
Q49	-0.134	**0.141**	0.079	0.197	0.104	0.212
Q5	-0.079	-0.003	**0.348**	-0.008	0.111	0.866
Q10	-0.029	-0.036	**0.415**	-0.010	-0.058	0.969
Q22	0.135	-0.119	**0.264**	0.125	-0.107	0.539
Q26	-0.034	-0.055	**0.464**	-0.028	-0.081	0.949
Q27	-0.091	-0.075	**0.417**	-0.031	0.044	0.911
Q31	-0.019	0.134	**0.293**	-0.014	0.020	0.819
Q41	0.024	0.071	**0.272**	0.036	0.039	0.898
Q45	0.092	0.084	**0.284**	-0.070	0.031	0.791
Q11	-0.053	0.025	0.029	**0.322**	-0.050	0.938
Q12	0.084	0.114	-0.128	**0.226**	-0.061	0.561
Q17	-0.310	0.074	0.066	**0.247**	-0.033	0.363
Q23	-0.016	0.101	0.189	**0.109**	-0.002	0.203
Q30	0.163	0.046	-0.051	**0.212**	-0.074	0.551
Q33	-0.039	-0.046	-0.026	**0.408**	-0.009	0.974
Q34	-0.071	0.003	-0.138	**0.465**	0.036	0.895
Q35	0.033	-0.243	-0.099	**0.326**	0.127	0.553
Q36	0.061	-0.119	0.101	**0.163**	0.098	0.412
Q42	0.056	-0.044	-0.002	**0.241**	0.077	0.841
Q43	0.102	-0.014	0.134	**0.131**	0.039	0.362
Q50	-0.009	0.103	-0.076	**0.204**	-0.147	0.521
Q3	0.080	0.009	-0.008	0.016	**0.241**	0.895
Q4	-0.113	-0.281	0.136	-0.001	**0.374**	0.559
Q14	0.018	0.038	-0.034	0.019	**0.395**	0.979
Q15	0.142	-0.003	-0.138	-0.070	**0.433**	0.809
Q19	-0.152	0.031	0.147	0.002	**0.405**	0.782
Q28	-0.023	0.043	-0.066	0.002	**0.444**	0.967
Q46	0.047	0.163	-0.038	0.033	**0.211**	0.587

Factor Fit	0.712	0.726	0.773	0.845	0.807	0.765

It is found that 17 of the 50 items manifest fit indices less than 0.70.

The current analysis sought the best fitting oblique solution to the 5-subscale structure. As such it provides correlations between the factors and these are reported in table 3. These correlations are very large suggesting that the independence of the separate scales is open to question.

**Table 3:** Factor Correlation Matrix

	I	II	III	IV	V
I. Perception of teaching:	1.000				
II. Perception of teachers	0.821	1.000			
III. Academic self-perception	0.788	0.767	1.000		
IV. Atmosphere	0.842	0.768	0.824	1.000	
V. Social self-perception	0.738	0.653	0.737	0.743	1.000

## Discussion

The DREEM is undoubtedly a useful tool for appraising the educational climate in medical education and its widespread international use reveals the need for such a device. However, there has perhaps been an inadequate focus on establishing and maintaining it's psychometric credentials. The present study highlights two concerns that may need attention. Firstly, the internal consistency of the 5 scales is quite variable and, in this sample, appears rather low. Secondly, the construct validity (the basis for the 5 subscales) is not well supported. Both of these findings do appear to be consistent with the Portugese [28], Greek [29] and Swedish [35] studies cited above. Given that our findings are based on Irish medical students it is unlikely that these weaknesses can be attributed to translation factors. As a result it is clear that the putative 5-factor model proposed by the developers of the DREEM is not supported and may be in need of revision.

It may also be tempting to suggest that a shortened 33 item DREEM may be formed by jettisoning the 17 weakest items identified in our factor analysis. However, great care needs to be taken in adopting such a strategy. Firstly, the weakest items in an Irish sample may not be the same as those identified in another nationality and secondly, in removing items the underlying factor structure may change dramatically. In the Roff et al. paper [12] describing the development of the DREEM it is clear that the subscale structure was driven by a-priori theoretical reasoning. The fact that empirical data do not conform well with this model might suggest that either the items need to be reframed to fit the model or that the model itself needs to be reconsidered. There is not yet sufficient published psychometric analysis across nationalities on the DREEM to suggest which is the most beneficial route to take in this regard.

Indeed, our finding showing very high correlations between the subscale factors may support the contention that the DREEM is essentially a one-factor single scale instrument. There is very little discrimination evident between the 5 subscales and reports of the DREEM do not typically examine the differential validity of the subscales.

## Conclusion

DREEM enjoys widespread usage as an instrument which measures the educational environment however factor analysis and subscale factor analysis raise questions about its basic psychometric properties and construct validity. These issues need to be addressed if DREEM continues to be used. Certainly, the latent model upon which it is built may need to be radically revised. This may be best achieved by a full integration of the existing multi-national exploratory analyses of the DREEM structure to inform a new empirically based latent model. This might then be followed by a large scale international sample being subjected to a full Structural Equation Modelling analysis. It is hoped that users of the DREEM will provide basic psychometric appraisal of the device in future published reports so that a more generalized picture of its cross-national viability becomes available.

## Competing interests

The authors declare that they have no competing interests.

## Authors' contributions

SM drafted the manuscript, participated in design of the study and carried out the analysis. MO'R participated in design and coordination was also responsible for data collection. MK participated in the design of the study. DB participated in the design of the study. SO'F conceived the study and participated in the design and coordination. All authors read, revised and approved the final manuscript.

## Pre-publication history

The pre-publication history for this paper can be accessed here:

http://www.biomedcentral.com/1472-6920/12/2/prepub
